# Supplementary figures and images for: Muscle strength gains per week are higher in the lower-body than the upper-body in resistance training experienced healthy young women—A systematic review with meta-analysis
Source: PLoS One. 2023 Apr 13;18(4):e0284216. doi: 10.1371/journal.pone.0284216 (PMC10101404; doi:10.1371/journal.pone.0284216)

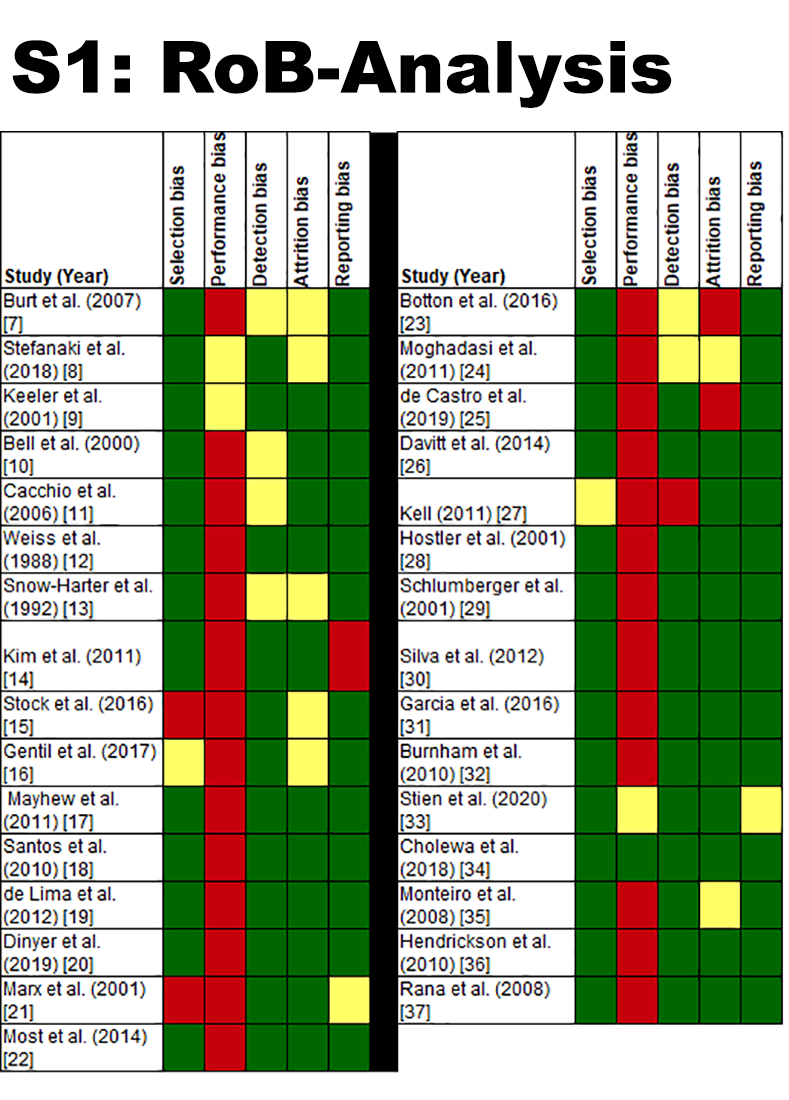

Supplement: S1 Appendix — (TIF) [file pone.0284216.s002.tif]
